# Optimized sample buffer for dispersed, high-resolution capillary zone electrophoretic separation of *Escherichia coli* B

**DOI:** 10.1038/s41598-023-49669-y

**Published:** 2023-12-14

**Authors:** Bonnie Jaskowski Huge, Caitlin M. Kerr, Sacheela Wanigasinghe, Matthew M. Champion, Norman J. Dovichi

**Affiliations:** 1 Department of Chemistry and Biochemistry, University of Notre Dame, Notre Dame, IN 46556 USA; 2 Berthiaume Institute for Precision Health, University of Notre Dame, Notre Dame, IN 46556 USA

**Keywords:** Microbiology, Analytical chemistry, Bioanalytical chemistry, Microfluidics

## Abstract

Capillary zone electrophoresis (CZE) is a powerful tool for high resolution chemical separations. Applying CZE to microbial samples may facilitate a deeper understanding of bacterial physiology and behavior. However, the study of complex microbial samples has been limited by the uncontrolled hetero-aggregation of bacterial cells under an applied electric field. We tested a wide range of sample buffers and buffer additives for the optimization of bacterial CZE separations using a 20 mM Tris–HCl background electrolyte. By modifying the sample buffer, but not the background electrolyte, we retain constant separation conditions, which aids in the comparison of the sample buffer additives. We report optimized methods for automated CZE separation and simultaneous fractionation of *Escherichia coli* B, which is one of the two most widely used wild-type strains. A modified sample buffer containing neutral salts and the addition of glycerol produced a 20-fold increase in loading capacity and a reduction in peak width/broadening of 86% in comparison to previously reported work. In addition, the glycerol-modified sample buffer appears to reduce the persistent aggregation and adhesion to the capillary walls during electrophoretic separations of complex environmental microbiota.

## Introduction

Experiments in the twentieth century investigated the behavior of bacteria in an electric field and during electrophoresis. These experiments were primarily used to measure the zeta potential of the microbes^1–8^. Late in the twentieth century, a small community of researchers investigated the use of capillary electrophoresis for microbial characterization. Ebersole and McCormick performed a pioneering study that demonstrated the use of capillary zone electrophoresis (CZE) with optical detection for the separation of two pure bacterial cultures^9^. Fractions were manually collected at the peak maximum.

Armstrong and colleagues reported higher resolution polymer-assisted CZE separation of microorganisms^10^. The charged surface of a fused silica capillary can invite bacterial adhesion, leading to reduced mobility or complete immobilization. Armstrong’s polymer-assisted separations were inspired by the desire to reduce interaction of the bacteria with the capillary wall. Unfortunately, electrostatic interaction between cells were reported to cause hetero-aggregation of bacterial species cells under an applied electric field in the presence of polymers^11,12^, resulting in irreproducible separations^13–16^.

We reported the coupling of a capillary electrophoresis system with an ink-jet printer-based system that deposited nanoliter fractions onto a petri dish with agar growth medium^17^. We employed the system to study separations of *E. coli* HB101 expressing green fluorescent protein (GFP). After incubation, the petri dish was observed under black light, and the separation was visually evaluated. The first grown-spot from each experiment were much more intense than subsequent spots, which formed a low-intensity tail.

*E. coli* HB101 has undergone a series of genetic manipulations and is a commonly used model organism for molecular biology applications. There are a number of other common *E. coli* strains used in research and industrial settings, as well as pathogenic strains that are of public health concern. In this paper, we report electrophoretic behavior of *E. coli* B, which is a wild-type strain commonly used for molecular biology and bacteriophage applications. We also consider the effect of a number of additives to the sample buffer. We optimized the sample buffer to contain neutral salts, non-ionic additives, and some chemical masking/bulking agents (e.g. polyamines, salinization reagents). Optimization of neutral salts and addition of glycerol resulted in a 20-fold increase in loading capacity and a reduction in peak tailing by 86% (measured from width of cultured migrated colonies) in comparison to an unmodified sample buffer. Trehalose, a nonreducing sugar, also performed well. We further demonstrate that the reduced apparent peak widths reflect improvement in separation efficiency and not simply recovery of aggregated cells by repeating the separation and determining the electrophoretic behavior of *E. coli* spiked into a complex environmental microbiota.

## Experimental

### Reagents and materials

Except as noted, reagents were analytical grade and purchased from Sigma-Aldrich (St. Louis, MO, USA). Solutions were prepared with deionized-distilled water (ddH_2_O) from a Barnstead Nanopure System (Thermo-Fisher Scientific, Waltham, MA USA). Microbial culture consumables and glassware were purchased sterile (Corning, Corning NY, USA, and VWR, Radnor, PA, USA) or autoclaved prior to use.

### Bacterial culture

Wild-type *E. coli* B was obtained from Ward’s Science (Rochester, NY, USA) and used to develop an electrolyte system for electrophoretic separations of intact microbial cells. After obtaining single colonies on LB agar, bacteria were subsequently cultured using LB medium (Teknova, Hollister, CA, USA) in culture tubes at 37 °C at 200 rpm overnight. Fresh LB medium was inoculated with the overnight culture (1:100 dilution) in shaking flasks and incubated at 37 °C at 250 rpm until they reached mid-exponential growth (OD_600_ ≈ 0.3). After growth, cultures were harvested by centrifugation (5000×*g*, 12 min, 4 °C) and washed with sterile-filtered PBS (Dulbecco’s phosphate-buffered saline). Washed cells were resuspended in sample buffer for subsequent analysis. Concentrations of cell suspensions were estimated by OD_600_ and verified by spot titers on LB plates.

*E. coli* B was transformed using a commercially available green fluorescent protein (GFP) expression vector, pGLO (Bio-Rad, Hercules, CA, USA) containing an arabinose inducible promoter pBAD, according to the manufacturer’s protocol to generate *E. coli* B:pGFP. Briefly, *E. coli* B cells were made electro-competent by repeated washings with 10% glycerol, followed by electroporation of pGLO into the competent cells using a MicroPulser™ (Bio-Rad) in a 0.1 mm cuvette. Selection was performed with ampicillin (100 µg/mL).

### Environmental sampling

Wastewater was sampled from a primary effluent tank at a local wastewater treatment plant (Mishawaka, Indiana). Microorganisms were isolated by centrifugation, washed, and suspended in PBS with glycerol (22%) for long-term storage at − 80 °C as described^17^. Aliquots were thawed and resuspended in sample buffer spiked with *E. coli* B:pGFP prior to analysis.

### Sample buffer and dispersants

Sample buffer was prepared at a final concentration of 36 mM Tris-base, 60 mM TAPS (3-(Tris(hydroxymethyl)methylamino)propane-1-sulfonic acid, A17754-22, Alfa Aesar, Ward Hill, MA, USA) without dispersant (control) and supplemented with dispersant at concentrations listed in Table 1. The following dispersants were evaluated as additives to the sample buffer: glycerol (G5517), d-(+)-trehalose dihydrate (T9531), antifoam 204 (A8311), picrylsulfonic acid solution (TNBS, P2297), (3-chloropropyl)trimethoxysilane (CPTMS, 440183), spermidine (85558), iodoacetic acid (IAA, 00652) from Sigma-Aldrich; Tween 80 (M126) from VWR Chemicals; Pluronic F-68 non-ionic surfactant (24,040,032) and bovine serum albumin (BSA, B14) from Thermo-Fisher Scientific; mineral oil from Meijer Distribution Inc (Walker, MI USA); and Proteinase K (KB-0111) from Bioneer (Oakland, CA USA).

**Table 1:** List of dispersants tested to determine influence on separation efficiency of *E. coli* B.

Dispersant	Concentration	References
Trehalose	20% (w/v)	^28,29,52–54^
Glycerol	22%; 0.1%, 1%, 10%, 20% (v/v)	^28,30,52–55^
Pluronic F-68	0.1% (v/v)	^28,31,32^
Bovine serum albumin (BSA)	0.1% (w/v)	^28,33^
Tween 80	0.01% (v/v)	^28,34–36^
Proteinase K	1 mg/mL	^39,40^
Mineral oil	0.05% (v/v)	^42^
Antifoam 204	0.1% (v/v)	^43,44^
(3-Chloropropyl)trimethoxysilane (CPTMS)	0.5% (v/v)	^45^
Picrylsulfonic acid solution (TNBS)	0.5% (v/v)	^46^
Iodoacetic acid (IAA)	10 mM, 25 mM	^47^
Spermidine	10 µM	^48–51^

A modified protocol was required for bacterial cell suspensions in sample buffer supplemented with proteinase K: Chloramphenicol was added to the cell suspension at a final concentration of 10 µg/mL and the solution was incubated at 37 °C for 1 h to activate proteinase K. Chloramphenicol, a bacteriostatic agent, was added to the suspension to arrest growth during incubation^18,19^.

### Instrumentation

The electrophoresis system was equipped with a 30 cm long bare fused capillary (50 μm ID, 150 μm OD, Polymicro Technologies, Phoenix, AZ, USA) that was inserted into an injection block^20^ supplied with high voltage for electrophoresis (CZE1000R, Spellman, Hauppauge, NY, USA).

We interfaced the capillary electrophoresis system with an automated fraction collector as reported^17^. The basic instrument is described in detail elsewhere^21^. Briefly, the distal end of the capillary was threaded into a Tee fitting (Upchurch Scientific, Oak Harbor, WA USA). The tip of the capillary was positioned at the exit of a dispensing nozzle (The Lee Company, Westbrook, CT USA). The nozzle was secured above a motorized stage (Prior Scientific, Rockland, MA USA) for deposition of fractions onto a collection plate. The instrument was controlled with software written in LabVIEW.

### Electrophoretic fractionation

The separation capillary was conditioned and rinsed with 1 M hydrochloric acid (HCl), 1 M sodium hydroxide (NaOH), ddH_2_O, and 20 mM Tris–HCl (10 mM Tris-base, 10 mM Tris–HCl, pH 8.2) in series prior to each analysis. The reservoir and lines supplying deposition buffer to the dispensing nozzle were flushed with 20 mM Tris–HCl at the beginning of each experiment.

For all experiments, the electrophoretic background electrolyte (BGE) and deposition buffer were matched (20 mM Tris–HCl). Samples were injected for 0.4 s at 4 psi. Electrophoresis and fractionation began simultaneously. Electrophoresis was performed at 400 V/cm, the deposition buffer was held under nitrogen pressure at 4 psi, and the nozzle was held at ground potential. The collection plate was secured to the motorized stage, which was programmed to move in a serpentine pattern^17^.

### Culture-dependent analysis

Electrophoretic fractions were collected directly onto LB agar. The instrument was programmed to move a distance of 5 mm after each deposition to generate a 12 × 12 grid of 144 fractions on the surface of the petri dish. The time interval between each deposition was 3.9 s. The volume of buffer dispensed with each fraction was approximately 1 µL. After each run, the petri dish was removed from the motorized stage, covered with a sterile lid, and allowed to dry at room temperature. Once dry, the plate was inverted and incubated at 32 °C overnight (~ 15 h). Plates were imaged for further analysis.

Fractions from electrophoretic fractionation of samples containing transformed *E. coli* B:pGFP were collected directly onto LB agar supplemented with l-arabinose to 0.2% (Teknova) (v/v). Plates were imaged under a UV lamp after incubation to visualize colonies expressing GFP.

## Results and discussion

Our preliminary investigation of the electrophoretic fractionation of bacteria used *E. coli* HB101^17^. This laboratory strain has significant genetic alterations that aid in its use as a model organism in molecular biology. In particular, HB101 lacks adhesins, which are proteins that mediate cell adherence to surfaces^22–25^. In this paper, we investigate the capillary electrophoretic behavior of wild-type *E. coli* B, and the effect of various sample buffer modifiers on the separation.

**Figure 1:**
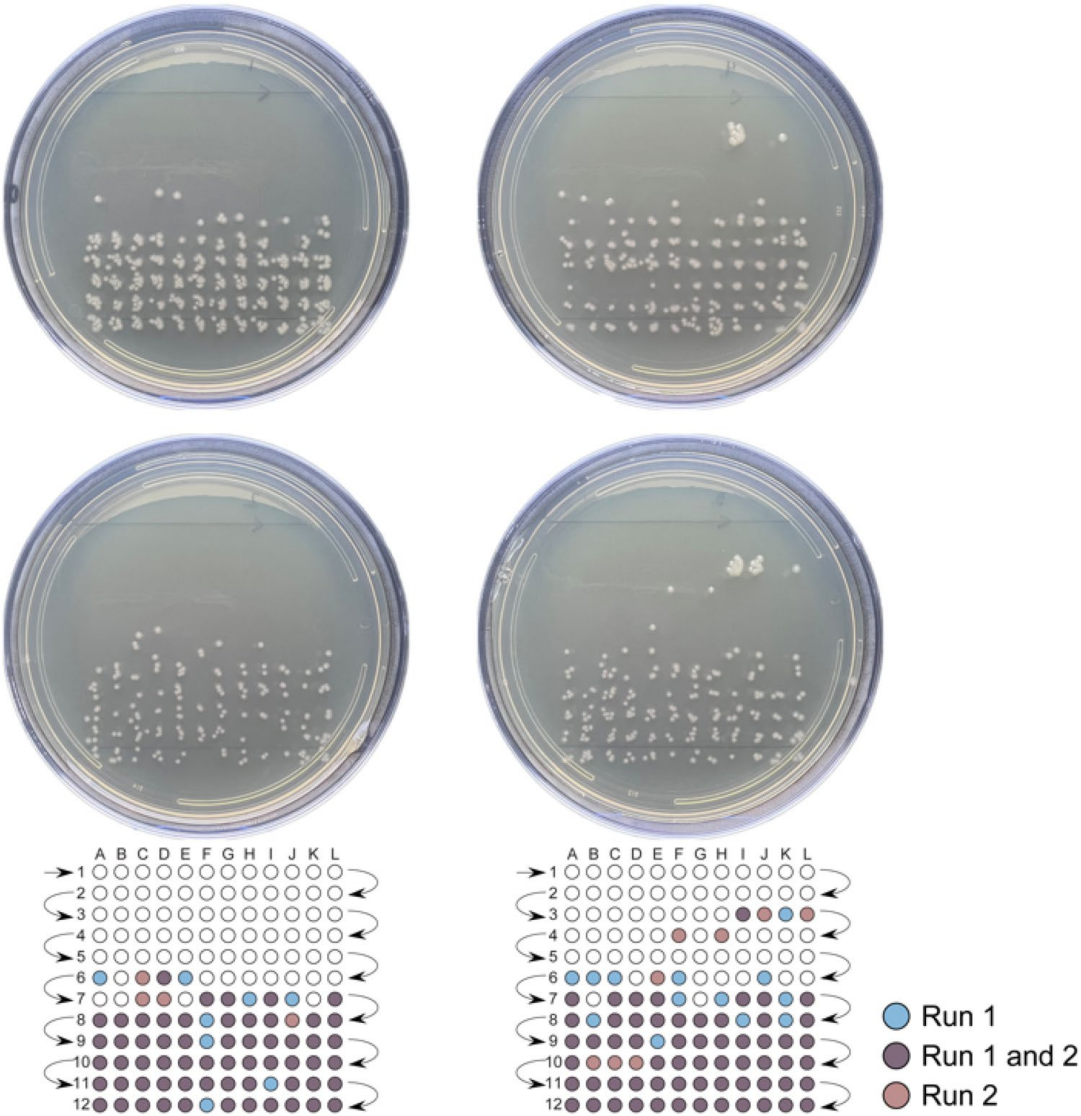
Comparison of *E. coli* B colonies after duplicate electrophoretic fractionation using matched and unmatched sample and separation systems. Approximately 1000 cells suspended in background electrolyte (20 mM Tris–HCl) (left), and 1000 cells suspended in a Tris-TAPS buffer (36 mM Tris-base, 60 mM TAPS) (right), were loaded by hydrodynamic injection and collected onto LB agar (fraction width: 3.9 s) in duplicate. Plates were incubated at 32 °C for 15 h and imaged. Reference grids are provided below each pair of images to highlight the location of colony forming units (CFUs).

Figure 1 presents duplicate fractionations of wild-type *E. coli* B cells (top and bottom). The background electrolyte for all runs was 20 mM Tris–HCl. Cells were viable after electrophoretic fractionation in a system where background electrolyte (BGE) and sample buffer were matched (Fig. 1, left). There was no obvious peak formed in this matched buffering system. The wild-type *E coli* B has much worse electrophoretic behavior than the laboratory strain HB101^17^.

In the unmatched system, the background electrolyte was also 20 mM Tris–HCl. However, *E. coli* B cells were suspended in 36 mM Tris-base, 60 mM TAPS sample buffer (Fig. 1, right). We used the zwitterionic buffering agent, TAPS, from Good’s biological buffers list^26^ in the sample buffer to maintain cell viability and to provide a reasonable balance in ionic strength between BGE and sample buffer^27^. This unmatched system recaptured some of the separation behavior of *E. coli* HB101. In the unmatched system, many of the cells migrate in a discrete zone at approximately 2.1 min. Though we have improved the separation of wild-type *E. coli* B using this unmatched buffering system, we note the persistence of trailing cells with migration after the main zone.

Bacteria can form aggregates in storage and under an applied electric field. These aggregates lead to irreproducible migration patterns^11–16^. In addition, cell adherence to the capillary walls would lead to significant band broadening.

We sought to improve both the separation efficiency and loading capacity of wild-type *E. coli* B cells by supplementing the sample buffer with a molecular dispersant. The list of dispersants used in this study is presented in Table 1. These dispersants were primarily chosen to reduce cellular aggregation, which degrades separation reproducibility. The dispersants should also reduce interaction of the cells with the capillary walls. Ideal dispersants would maintain cell viability and be of neutral charge. By modifying the sample buffer, but not the background electrolyte, we retain constant separation conditions, which aids in the comparison of the dispersants. The initial concentration of each dispersant was based on literature. Dispersants that improved separation efficiency were further tested at a range of concentrations.

We investigated five common cryoprotectants: trehalose, glycerol, Pluronic F-68, Tween 80, and bovine serum albumin (BSA)^28^. Both trehalose and glycerol are hydrophilic polyols that have a protective effect on cell membranes^28,29^. It is reported that trehalose is highly effective in maintaining microbial viability, but its use is limited in practice due to its relatively high cost. Stefanello et al*.* observed direct correlation of microbial survivability and trehalose concentration when used as a cryoprotectant, but reagent costs prevented them from confirming this trend at concentrations above 30%^29^. Glycerol is a highly effective and low-cost alternative cryoprotectant used to stabilize bacteria in the concentration range of 2–55%^28,30^. Initial concentrations tested for effects on electrophoretic separation efficiency of *E. coli* B were 20% trehalose and 22% glycerol (v/v).

Pluronics are triblock copolymers consisting of hydrophilic poly(ethyleneoxide)-poly(propyleneoxide)-poly(ethyleneoxide): PEO-PPO-PEO triblock copolymer. PEO is among the nonpenetrating or nonpermeating compounds used in microbiology as an extracellular cryoprotectant at concentrations from 5 to 15%^28^. Pluronic F-68 is a non-ionic surfactant used to control shear forces in suspension cultures. It can also be used to reduce foaming and limit adhesion to glass. Pluronic F-68 is reported as an effective cell protectant in culture at concentrations in the range of 0.01–1%^31^. These copolymers self-assemble into micelles in aqueous solutions, suggesting potential application as pseudo-stationary phases in micellar electrokinetic chromatography^32^. Initial experiments used the manufacturer’s suggested working concentration of 0.1% (v/v).

BSA is used for stabilization of enzymes and microorganisms during storage; serum albumins have been used as cryoprotectants at concentrations of 0.1–4%^28^. BSA interacts nonspecifically with bacteria and various surfaces resulting in decreased bacterial retention by reducing the adhesion strength of bacterial cells^33^. Electrophoretic mobility measurements of bacterial cells with and without BSA proteins revealed that the protein adsorbed on the cell wall of the two bacterial strains tested but did not saturate the bacterial surface even when the protein concentration was in excess^33^. Based on these observations, we applied BSA as a dispersant at a concentration of 0.1% (w/v).

Tween 80 has been widely used in biochemical applications as an emulsifying and dispersing substance in medicinal and food products. It has little or no activity as an antibacterial agent and serves as an effective cryoprotectant at concentrations as low as 1%^28,34^. Toutain-Kidd et al*.* show treatment of bacterial cells with 0.01% Tween 80 did not alter growth and resulted in a 100-fold reduction in attachment to PVC and glass surfaces^35^. These results were consistent with earlier work by Barkovskii et al*.* where treatment with 0.01% Tween 80 was able to disrupt environmental microbe-peat association^36^. We applied Tween 80 in our electrophoretic separations at a concentration of 0.01% (v/v), but even at this relatively low concentration, addition of Tween 80 to the sample buffer resulted in bubble formation, which is incompatible with capillary electrophoresis.

Biofilms are a community of bacteria living within a secreted matrix of extracellular polymeric substances (EPS), such as lipids, polysaccharides, proteins, and extracellular nucleic acids^37^. Relying on reagent interaction with major EPS components to disperse biofilms and cell suspensions^38^, we included the following dispersants: Proteinase K, mineral oil, Antifoam 204, (3-chloropropyl)trimethoxysilane (CPTMS), picrylsulfonic acid solution (TNBS), iodoacetic acid (IAA), and spermidine.

Proteinase K targets proteins that contribute to biofilm formation. Shukla et al*.* report proteinase K treatment in the range of 2–32 µg/mL did not inhibit planktonic growth of *Staphlococcus aureus*, and a concentration of just 2 µg/mL caused significant dispersal of pre-grown *S. aureus* biofilms^39,40^. Proteinase K is effective at concentrations ranging from 0.01 to 1 mg/mL with incubation in the range of 37–55 °C; optimal incubation is at 37 °C^41^. Given its low toxicity and reported effectiveness as a dispersant, we applied 1 mg/mL proteinase K with incubation at 37 °C for one hour to disrupt any natural aggregation of bacterial cells; apparent toxicity remained negligible at 1 mg/mL. However, addition of proteinase K to the sample buffer proved incompatible with capillary electrophoresis due to excessive foaming.

Mineral oil promotes dispersion of microbial cells by interacting with lipids on the cell surface^42^. Mineral oil was tested at a relatively low concentration of 0.05% (v/v).

Antifoam reagents are common in culture media to control foaming and minimize damage to cells. Cho et al*.* report its application more specifically to prevent bubble formation due to aeration in LB culture medium for bacterial growth^43^. Antifoam 204 is a synthetic mixture of organic non-silicone polypropylene-based polyether dispersions that affects the surface tension of the culture^44^. Antifoam 204 was initially tested at 0.01% (v/v) (within range of manufacturer’s recommendation, data not shown). Based on preliminary results, we increased the concentration to 0.1% (v/v).

CPTMS is an organo-silane that forms a self-assembled monolayer, which facilitates the modification of different surfaces. This non-interactive neutral coating prevents bacterial cell adhesion^45^. We assessed CPTMS at a concentration of 0.5% (v/v).

TNBS is a non-permeating hapten that interacts with surface amine groups. Bogdanov et al*.* applied TNBS as a nonpenetrating membrane probe in their study of lipid dynamics on the outer membrane of *E. coli* cells^46^. Their results indicated that TNBS bound to all available surface-accessible amino groups. We applied TNBS at a concentration of 0.5% (v/v) to disperse bacterial cells by coating the outer membrane, reducing cell–cell and cell-surface interactions.

IAA is an alkylating agent that covalently alkylates sulfhydryl moieties (-SH) including cysteine. In development of a potential prodrug system for wound disinfection, tests by Weng et al*.* demonstrate that IAA possesses good biocompatibility and negligible toxicity to *S. aureus* suspensions and biofilms subjected to treatment^47^. We tested the effects of IAA on *E. coli* B at concentrations of 10 mM and 25 mM. At concentrations greater than 25 mM, our results indicated that the toxicity of IAA is no longer negligible for *E. coli* suspensions (data not shown).

Finally, we applied spermidine to enhance separation efficiency at a final concentration of 10 µM. Spermidine is a polyamine routinely used in DNA purification and we hypothesized that it might mitigate aggregation from extracellular nucleic acids^48^. Chattopadhyay et al*.* observed reduced growth rates in spermidine-deficient *E. coli* (40–50%) and profound morphological changes in yeast grown in spermidine-deficient medium^49,50^. Addition of spermidine may also provide nutrient support in a stressful environment^51^.

### Assessing the dispersants' influence on separation efficiency

**Figure 2:**
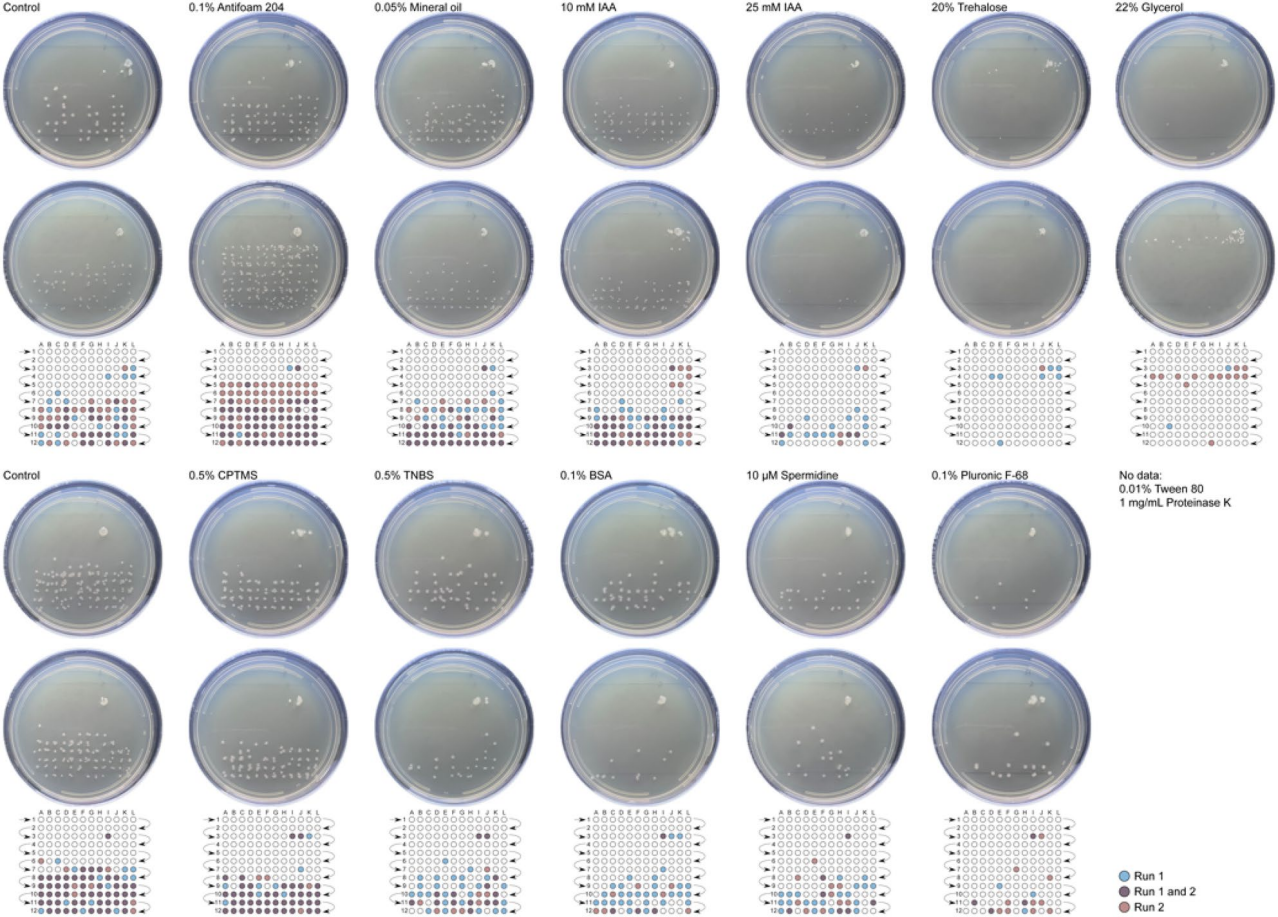
Images of *E. coli* B colonies after electrophoretic fractionation used to determine the optimal dispersant. Approximately 1200 cells (top) and 600 cells (bottom) were loaded by hydrodynamic injection and collected onto LB agar (fraction width: 3.9 s) in duplicate. *E. coli* B were resuspended in sample buffer (control) and sample buffer supplemented with dispersants as listed in Table 1 and above. Plates were incubated at 32 °C for 15 h and imaged. Reference grids are provided below each set of images to highlight the location of CFUs.

To evaluate the effect of each dispersant listed in Table 1, we generated a series of samples from an initial culture of *E. coli* B grown to a concentration of ≥ 2 × 10^9^ cells/mL; *E. coli* B cells were cultured, washed, and resuspended in sterile PBS. The cell suspension was then aliquoted and diluted in sample buffer (control) and sample buffer supplemented with dispersant to produce a final concentration of approximately 1.8 × 10^8^ cells/mL for a total injection of approximately 1000 cells. Samples were injected, separated, and deposited onto solid growth media: this process was completed in duplicate for each sample. The location and density of the resulting colony forming units (CFUs) from each run were evaluated to measure the effect of the dispersant on separation efficiency relative to the control shown in Fig. 2.

Figure 2, top, from left to right: relative to control, addition of Antifoam 204 did not improve separation efficiency of *E. coli* B. We also observed negative effects, to a lesser degree, on separation efficiency when *E. coli* B were suspended in sample buffer supplemented with mineral oil or 10 mM IAA prior to injection. Increasing the concentration of IAA from 10 to 25 mM IAA trended toward improved separation efficiency. At concentrations greater than 25 mM, our results indicated that IAA becomes toxic to *E. coli* B cells in suspension (data not shown). Addition of trehalose or glycerol resulted in the most obvious and profound effects on separation efficiency of *E. coli* B.

Figure 2, bottom, from left to right: relative to control, addition of CPTMS, TNBS, BSA, Spermidine, and Pluronic F-68 show moderate improvement in separation efficiency of *E. coli* B with the most significant improvement in the presence of Pluronic F-68. Addition of Tween 80 or proteinase K to the sample buffer proved incompatible with capillary electrophoresis; no results were generated from these experiments.

Microorganisms naturally produce protective polyols as a stress-response to certain environmental changes^52–54^. Managbanag and Torzilli observed the cellular concentrations of trehalose and glycerol increase in heat- and/or salt-stressed cells^54^. These observations correspond to earlier reports concerning osmotic shock response^53^. We assume that electrophoresis produces a similar stress-response in bacterial cells and correlates well with the data generated from electrophoretic fractionation of samples supplemented with 20% trehalose or 22% glycerol.

Figure 2 provides strong evidence in support of supplementing the sample buffer with either trehalose or glycerol for high separation efficiency. In addition to biocompatibility, glycerol has been shown to be a highly effective additive in CZE separations of serum samples. Pascali et al*.* provide data to support supplementing BGE with glycerol in the range of 10–30% (v/v), where increasing the percentage of glycerol in BGE was paralleled by an increase in separation resolution for quantification of bromide in serum^55^.

**Figure 3:**
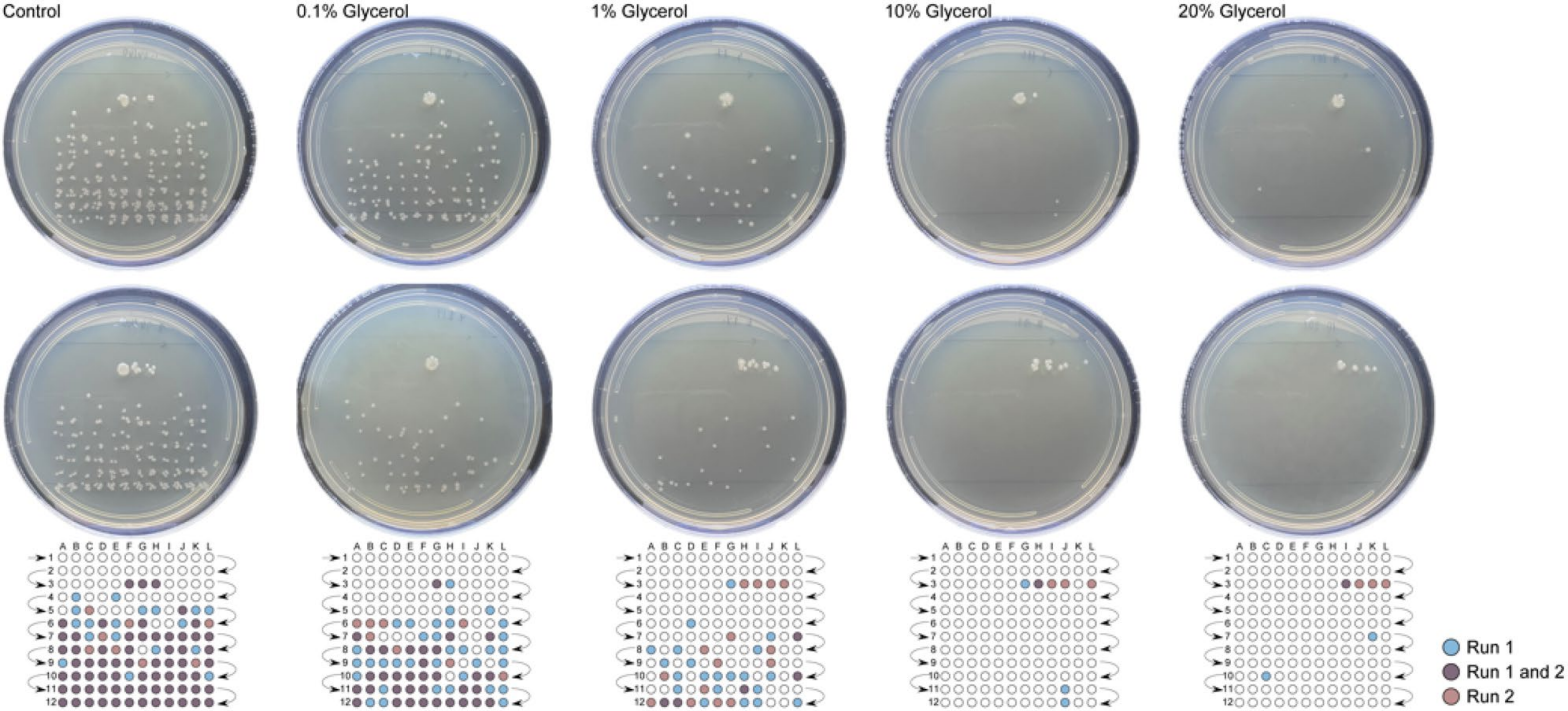
Images of *E. coli* B colonies after electrophoretic fractionation used to determine the optimal dispersant concentration. Approximately 1000 cells were loaded by hydrodynamic injection and collected onto LB agar (fraction width: 3.9 s) in duplicate. *E. coli* B cells were resuspended in sample buffer supplemented with glycerol at various concentrations, from left to right: 0% (control), 0.1%, 1%, 10%, 20%. Plates were incubated at 32 °C for 15 h and imaged. Reference grids are provided below each set of images to highlight the location of CFUs.

We next investigated the influence of glycerol at concentrations ranging from 0.1 to 20% (v/v) on separation efficiency. Sample injection amount was held constant at 1000 cells for all glycerol concentrations. The location and density of the resulting CFUs from each run were evaluated to measure the effect of varying the concentration of glycerol on separation efficiency relative to the control. Figure 3, from left to right: separation efficiency increased with increasing concentrations of glycerol.

**Figure 4:**
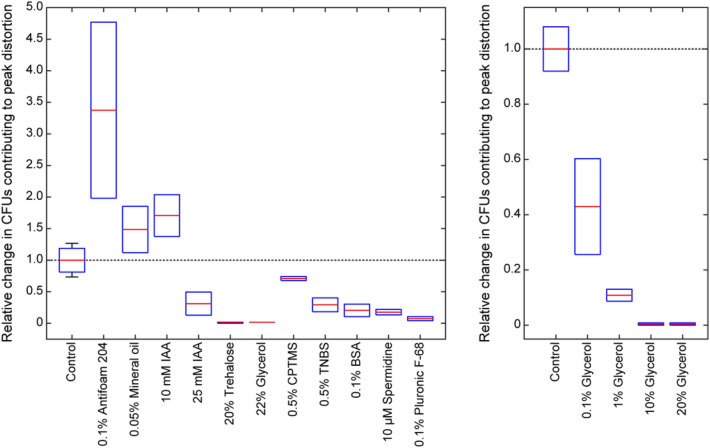
Box plots of the relative change in the number of cells with migration after the main zone in sample buffer supplemented with dispersant as compared to control. Colonies were considered to be contributing to peak distortion if they migrated after the main peak by ≥ 1 min. Relative change in separation efficiency was calculated by dividing the number of CFUs contributing to distortion in sample buffer supplemented with dispersant by the number of CFUs contributing to distortion in the control. Plots were generated from results presented in Fig. 2 (left) and Fig. 3 (right).

We calculated relative change in the number of cells with migration after the main zone to assess the dispersant data in total and to determine the optimal conditions for high resolution electrophoretic fractionation of *E. coli*. Control and dispersant data are displayed as box plots in Fig. 4. Each box plot represents the minimum, maximum, interquartile range, and median output from the following equation:

$$Relative \,\, change=\frac{\# CFUs \,\,distorting \,\,peak \,\,in \,\,dispersant}{\# CFUs \,\,distorting \,\,peak \,\,in \,\,control \,\,(replicate \,\,average)}$$

where the number of CFUs contributing to peak distortion (CFUs formed in rows 5–12) were visually counted. A proportional version of the analysis presented as Fig. 4 is provided in Fig. S1, where we considered only presence or absence of CFUs in rows 5–12. From these data, we conclude supplementing the sample buffer with 20% glycerol is optimal for high resolution electrophoretic separation of *E. coli* B.

The volume injected, and therefore the number of cells injected, could be influenced by sample buffer properties such as viscosity. Theoretical injection volume calculations that account for the difference in viscosity when the sample buffer is supplemented with glycerol are summarized in Table S1. We compared the effect of viscosity on the quantity of sample injected in two additional experiments.

First, we employed a laser-induced fluorescence detection system for analysis of constant injection parameters using a fluorescent standard in sample buffer (control) and sample buffer supplemented with 10% or 20% glycerol (Fig. S2). The laser-induced fluorescence detection system is described in detail in ref^20^. Identical injection conditions were applied for each sample buffer composition. Peak area for control data were not significantly different from samples supplemented with glycerol. These data demonstrate that the injected sample volume is consistent and independent from changes in fluid properties due to addition of glycerol in the range of 0–20%.

Next, we applied the corrected injection parameters to *E. coli* B cells suspended in sample buffer (control) and sample buffer supplemented with 10% or 20% glycerol (Fig. S3). Injection parameters were corrected to normalize sample load based on the assumption that increasing solution viscosity reduces sample load. Quantifying these data is less straightforward, however it is evident that supplementing the sample buffer with glycerol results in superior resolution compared to control.

### Effects of dispersants on mixed-aggregate formation under applied electric field

**Figure 5:**
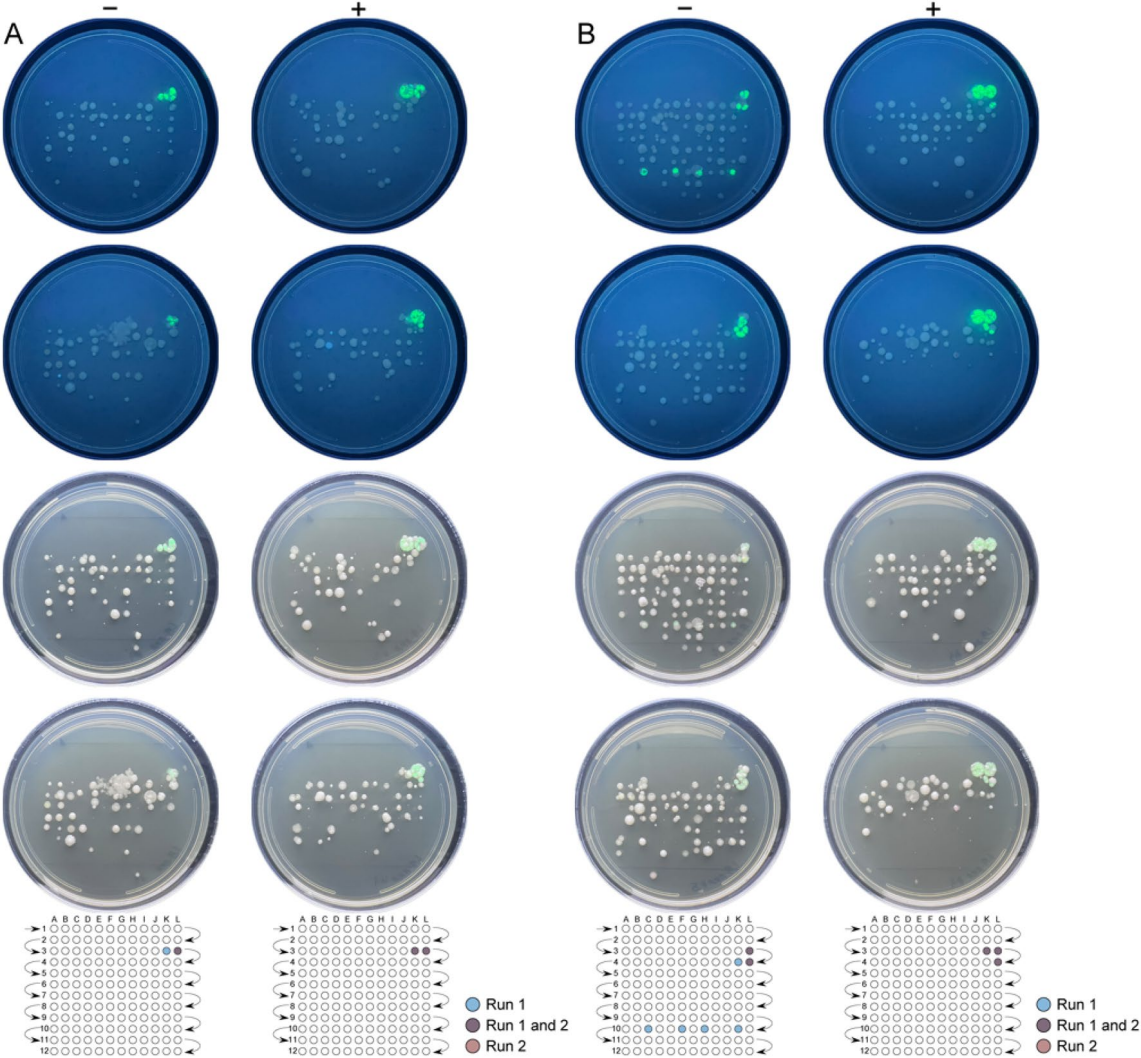
Addition of glycerol reduces hetero-aggregate formation in mixed microbial separations. Microorganisms isolated from wastewater were spiked with *E. coli* B:pGFP for an injection that contained 150 *E. coli* B:pGFP cells in 10,000 cells isolated from wastewater (**A**) and 1400 *E. coli* B:pGFP cells in 10,000 cells isolated from wastewater (**B**). Cell mixtures were loaded by hydrodynamic injection and collected onto LB-agar supplemented with L-arabinose (fraction width: 3.9 s) in duplicate. Cell mixtures were resuspended in sample buffer (control; columns marked with −) and sample buffer supplemented 20% glycerol (columns marked with +). Plates were incubated at 32 °C for 15 h and imaged (not shown). Plates were incubated at RT for an additional 24 h to visualize differences in colony morphology and imaged under a UV lamp to identify colonies formed by *E. coli* B:pGFP (top two rows). The same plates were also imaged under white light to observe culturable environmental microbes (bottom two rows). Reference grids are provided below each set of images to highlight the location of *E. coli* B:pGFP colonies.

In order to test that the addition of glycerol does not promote formation of mixed species aggregates (hetero/mixed-aggregates), we prepared environmental microbial samples for electrophoretic fractionation. Microorganisms isolated from wastewater were mixed with transformed *E. coli* B expressing GFP and suspended in sample buffer (control) and sample buffer supplemented with glycerol (20%). Images of *E. coli* B spiked-wastewater microbiota are presented in Fig. 5.

Figure 5 shows colonies formed after electrophoretic fractionation of an *E. coli* B:pGFP spiked wastewater microbiome sample. *E. coli* B:pGFP spiked samples were prepared for injection to contain 150 cells (Fig. 5A) and 1400 cells respectively (Fig. 5B) in a background of approximately 10,000 microbial cells isolated from wastewater. Each sample was suspended in sample buffer (control; columns marked with −) and sample buffer supplemented with 20% glycerol (columns marked with +). At a relatively low concentration of 150 cells (Fig. 5A), *E. coli* B:pGFP produced colonies in only 1 or 2 fractions with an average migration time of 2.3 min (control) and 2.2 min (20% glycerol). In Fig. 5B, where 1400 *E. coli* B:pGFP cells were injected, the control runs show *E. coli* B:pGFP cells migrated at 2.3 min with additional fluorescent colonies formed at 7.1–7.7 min. When supplemented with 20% glycerol, the *E. coli* B:pGFP cells produced dense colony growth at a migration time of 2.2 min. There is no evidence of tailing when the sample buffer is supplemented with glycerol.

CFUs lacking fluorescence derive from the culturable portion of the wastewater microbiota. Based on these data, addition of 20% glycerol to the sample buffer enhanced separation efficiency of *E. coli* B without promoting mixed-species aggregation under an applied electric field.

### Measuring improvement of optimized conditions

In order to measure the magnitude of the improvement from the addition of glycerol, we applied the modified sample buffer with 20% glycerol for the electrophoretic separation of *E. coli* B to the laboratory strain, *E. coli* HB101 (Supporting Information S1). The 20% glycerol sample buffer produced a significant increase in separation efficiency and loading capacity compared to previously published work^17^. Figure S4 compares *E. coli* HB101 colonies after electrophoretic fractionation using our previously reported protocol (A), where 2500 *E. coli* HB101 cells span a peak width of 66 ± 6 s, and our current protocol (B), where 50,000 *E. coli* HB101 cells span a peak width of 9 ± 3 s. Applying the methods described in this manuscript to electrophoretic separation of intact *E. coli* HB101, we achieved a 20-fold increase in loading capacity with peak width reduced by 86%.

*E. coli* HB101 is a laboratory strain that is derived from wild-type *E. coli* B^56^. Laboratory strains, such as HB101, are bioengineered derivatives and are traditionally employed for efficient DNA cloning in microbiology^57^. In recent work, HB101 has proven useful in studies concerning cell-surface and cell–cell adherence: Nascimento et al*.* employ *E. coli* HB101 as a non-adherent control since it lacks the surface structures that are associated with adhesion and biofilm formation^22,23^. We hypothesize that the degree of separation efficiency observed in electrophoretic analysis of *E. coli* HB101 results from its lack of cell surface structures; more specifically, we suspect the lack of surface structures that are directly responsible for motility and/or adhesion (i.e., flagella, fimbriae)^24,25,58–60^.

## Conclusions

Addition of glycerol to the sample buffer dramatically improves separation efficiency for *E. coli* B and *E. coli* HB101 compared with the use of background electrolyte for sample suspension. The glycerol appears to minimize bacterial adhesion to the capillary wall and to other bacteria.

The effect of the glycerol-modified sample buffer on the electrophoretic separation of a complex environmental microbiome is difficult to discern when relying on colony formation. Additionally, the role of biological *vs* electrophoretic aggregation/hetero-aggregation is not fully described and remains a barrier in the field. We are actively exploring experiments to differentiate these aggregation phenotypes. More sophisticated genetic sequence analysis will be required to explore the benefits of addition of dispersants to sample buffers in capillary electrophoresis analysis of complex environmental microbiomes.

We only considered the effect of reagents added to the sample buffer; the separation buffer was kept constant in all experiments. In contrast, Błońska and colleagues recently reported the use of five different separation buffers for the CZE separation and UV detection of two pure bacterial cultures with manual collection of fractions^61^. They reported that the best separations were produced using a 10 mM phosphate buffer supplemented with 0.1% Brij 35, 0.05% PEG, and 5% EtOH. We employed a simpler background electrolyte for our separations (20 mM Tris, pH 8.2). Since we match the deposition and separation buffers, the use of Błońska’s separation electrolyte would expose bacteria to ethanol during culture, which is not desirable for the complex environmental microbiome used in our experiments.

### Supplementary Information

Supplementary Information.

## Data Availability

All data generated or analyzed during this study are included in this published article (and its Supporting Information files S1).
