# Supplementary material for: Optimized sample buffer for dispersed, high-resolution capillary zone electrophoretic separation of Escherichia coli B
Source: Sci Rep. 2023 Dec 14;13:22269. doi: 10.1038/s41598-023-49669-y (PMC10721931; doi:10.1038/s41598-023-49669-y)
Supplement: Supplementary file 1 — Supplementary Information. [file 41598_2023_49669_MOESM1_ESM.docx]

**SUPPORTING INFORMATION**

**Optimized sample buffer for dispersed, high-resolution capillary zone electrophoretic separation of *Escherichia coli* B**

Bonnie Jaskowski Huge ^1⤉^, Caitlin M. Kerr ^1⤉^, Sacheela Wanigasinghe ^1^, Matthew M. Champion ^1,2*^,

and Norman J. Dovichi ^1,2*^

^1^Department of Chemistry and Biochemistry, and ^2^Berthiaume Institute for Precision Health,

University of Notre Dame, Notre Dame, IN 46556 USA

*email: [ndovichi@nd.edu](mailto:ndovichi@nd.edu), [mchampio@nd.edu](mailto:mchampio@nd.edu)

**Supporting Methods.** Modifications to methods for bacterial culture, instrumentation, electrophoretic fractionation, and culture-dependent analysis of *E. coli* HB101

**Figure S1.** Percentage of fractions occupied by CFUs with migration time ≥ 1 minute after the main zone (rows 5-12).

**Table S1.** Theoretical influence of sample buffer viscosity on hydrodynamic injection volume and injection time correction.

**Figure S2.** Electropherograms and peak area statistics generated from hydrodynamic injection of 10 nM fluorescein solutions, in triplicate, without correction for sample buffer viscosity.

**Figure S3.** Images of *E. coli* B colonies after triplicate electrophoretic fractionation with injection time corrected for sample buffer viscosity.

**Figure S4.** Comparison of *E. coli* HB101 colonies after electrophoretic fractionation using our previously reported protocol and our current protocol.

**Supporting Methods**

**Bacterial Culture**

*Escherichia coli* HB101 was obtained from Coli Genetic Stock Center (CGSC at Yale University). Bacteria were cultured using LB medium (Teknova) supplemented with 100 $\mu$g/mL ampicillin in culture tubes at 37 °C at 200 rpm overnight. Fresh LB medium supplemented with 100 µg/mL ampicillin was inoculated with the overnight culture (1:100 dilution) in shaking flasks and incubated at 37 °C at 250 rpm until they reached mid-exponential growth. After growth, cultures were harvested by centrifugation (5000xg, 12 min, 4°C) and washed with sterile-filtered PBS (Dulbecco’s phosphate-buffered saline). Washed cells were resuspended in sample buffer for subsequent analysis; PBS (**Figure S4A**) or 36 mM Tris-base, 60 mM TAPS (**Figure S4B**) supplemented with 22% glycerol. Concentrations of cell suspensions were estimated by optical density at 600 nm (OD_600_) and verified by spot titer.

**Instrumentation**

For results presented in **Figure S4A**, the electrophoresis system was equipped with a 60 cm long bare fused capillary (100 μm ID, 150 μm OD, Polymicro Technologies) that was inserted into an injection block supplied with high voltage for electrophoresis (Spellman CZE1000R). The electrophoresis system for **Figure S4B** is described in detail in the main text.

Capillary zone electrophoresis was interfaced with culture-based detection as described in the main text.

**Electrophoretic Fractionation**

The separation capillary was conditioned and rinsed with 1 M hydrochloric acid (HCl), 1 M sodium hydroxide (NaOH), ddH_2_O, and BGE in series prior to each analysis. The reservoir and lines supplying deposition buffer to the dispensing nozzle were flushed with BGE at the beginning of each experiment.

The BGE and deposition buffer were matched for each experiment; 10 mM Tris-HCl (10 mM Tris-base titrated with 1 M HCl, pH 8.0) for **Figure S4A** and 20 mM Tris-HCl (10 mM Tris-base, 10 mM Tris-HCl, pH 8.2) for **Figure S4B**. Samples were injected for 0.5 s at 3.5 psi for **Figure S4A** and 0.4 s at 4 psi for **Figure S4B**. Electrophoresis and fractionation began simultaneously. Electrophoresis was performed at 233 V/cm for **Figure S4A** and 400 V/cm for **Figure S4B**, the deposition buffer was held under nitrogen pressure at 4 psi, and the nozzle was held at ground potential. The collection plate was secured to the motorized stage, which was programmed to move in a serpentine pattern.

**Culture-Dependent Analysis**

Fractions from electrophoretic fractionation were collected directly onto LB agar. The instrument was programmed to move a distance of 5 mm after each deposition to generate a 12 x 12 grid of 144 fractions on the surface of the petri dish. The time interval between each deposition was 9.1 s for **Figure S4A** and 4.1 s for **Figure S4B**. The volume of buffer dispensed with each fraction was approximately 1 µL. After each run, the petri dish was removed from the motorized stage, covered with a sterile lid, and allowed to dry at room temperature. Once dry, the plate was inverted and incubated at 37 °C overnight (~15 hours). Plates were imaged for further analysis.


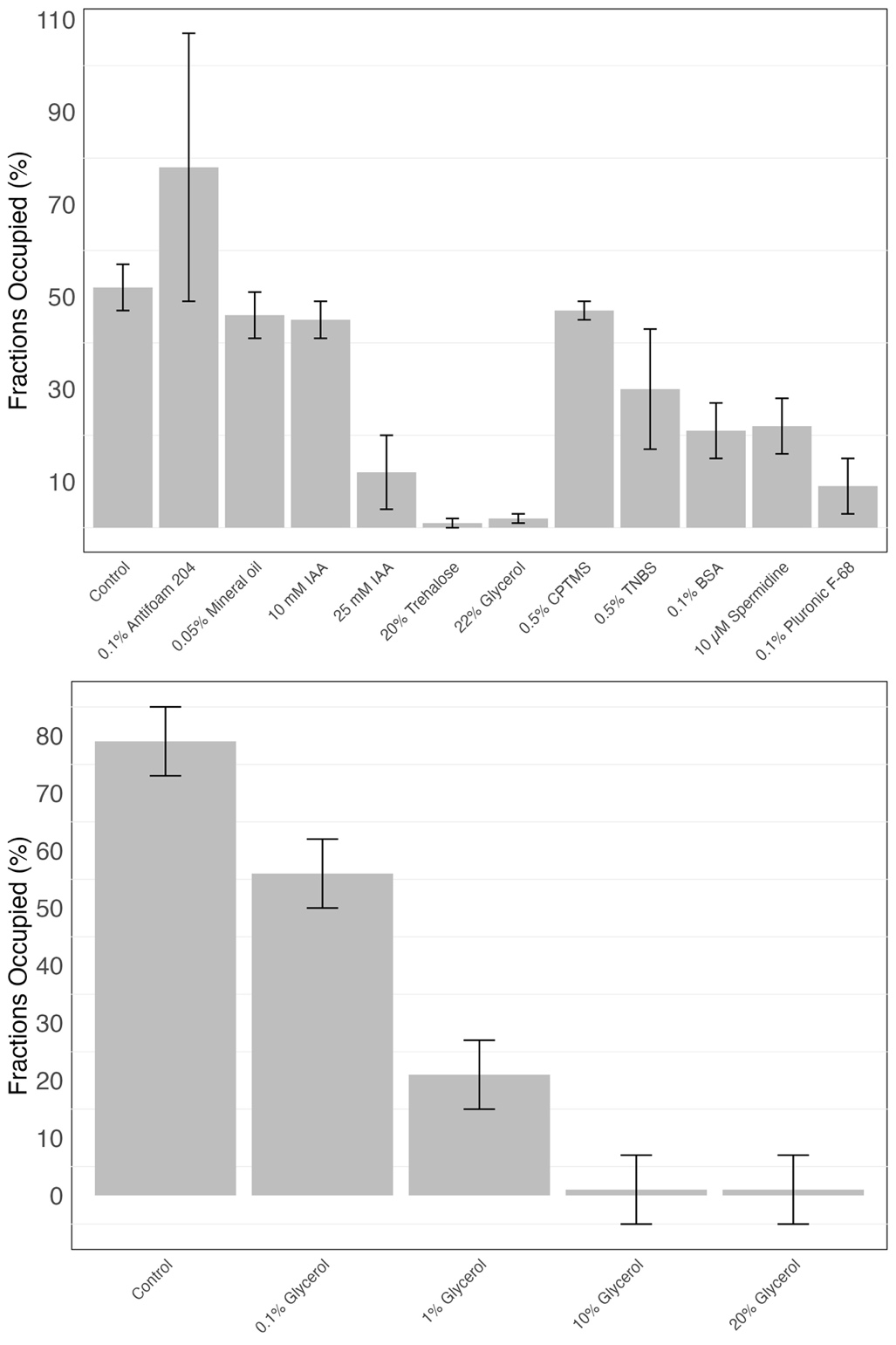


**Figure S1.** Percentage of fractions occupied by CFUs with migration time ≥ 1 minute after the main zone (rows 5-12). Percent of fractions occupied for each sample buffer composition were calculated by dividing the number of fractions containing CFUs in rows 5-12 by the total number of fractions in rows 5-12 (96). Error bars represent the standard deviation from the mean. Plots were generated from results presented in **Figure 2** (top) and **Figure 3** (bottom) in R.

**Table S1.** Theoretical influence of sample buffer viscosity on hydrodynamic injection volume and injection time correction

| ***Glycerol (%)*** | ***Injection time (s)*** | ***Viscosity (mPa·s)*** | ***Relative amount injected (%)*** | ***Corrected injection time (s)*** | ***Relative amount injected (%)*** |
| --- | --- | --- | --- | --- | --- |
| 0 | 0.4 | 1.0049 | 100 | 0.4 | 100 |
| 0.1 | 0.4 | 1.0079 | 100 | 0.4 | 100 |
| 1 | 0.4 | 1.0355 | 97 | 0.4 | 97 |
| 10 | 0.4 | 1.3415 | 75 | 0.5 | 94 |
| 20 | 0.4 | 1.7508 | 57 | 0.7 | 100 |

**
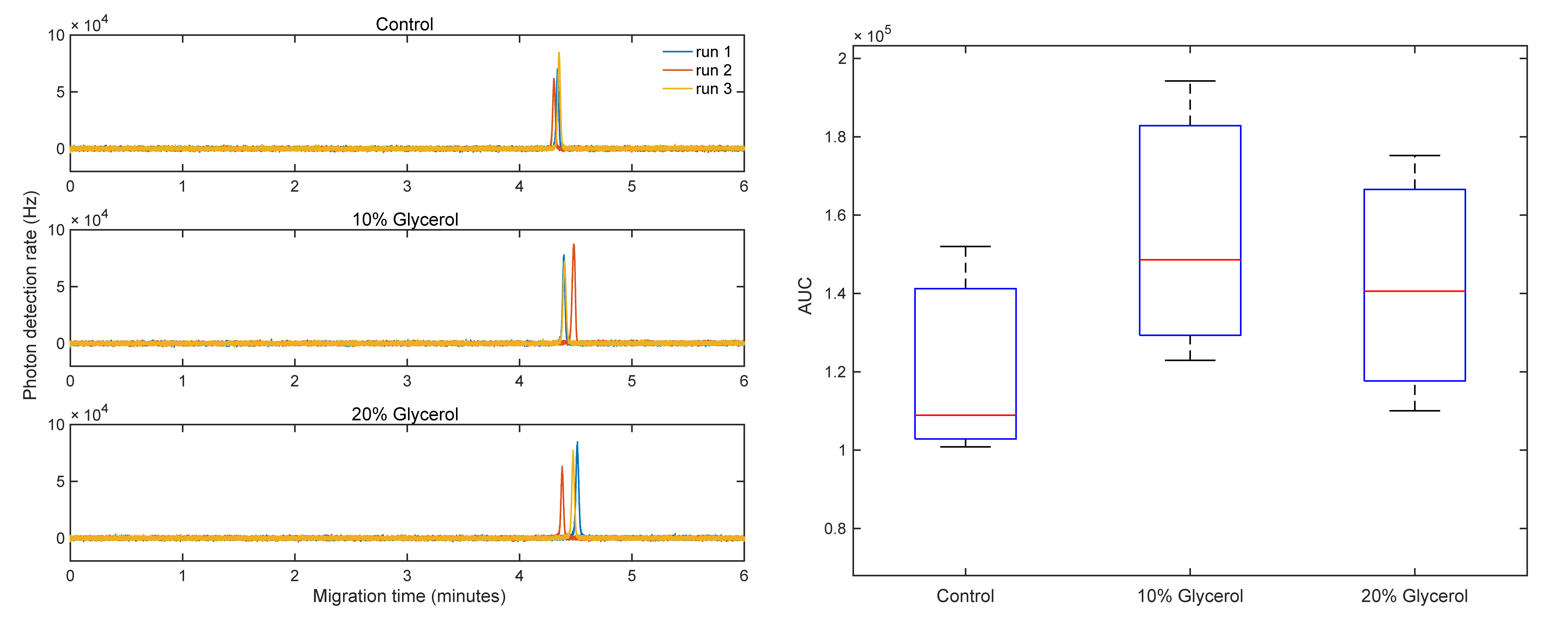
**

**Figure S2.** Electropherograms and peak area statistics generated from hydrodynamic injection of 10 nM fluorescein solutions, in triplicate, without correction for sample buffer viscosity. Signals reached saturation in the first detector. Attenuated signals from the next detector in series were analyzed and the results are displayed. (Left) Replicate traces are overlaid and color coded for each sample buffer composition: control (0% glycerol), 10% glycerol, 20% glycerol. (Right) Replicate peak areas are displayed as boxplots: control was not significantly different from either treatment (student’s t-test, p-value > 0.05, n=3).

**
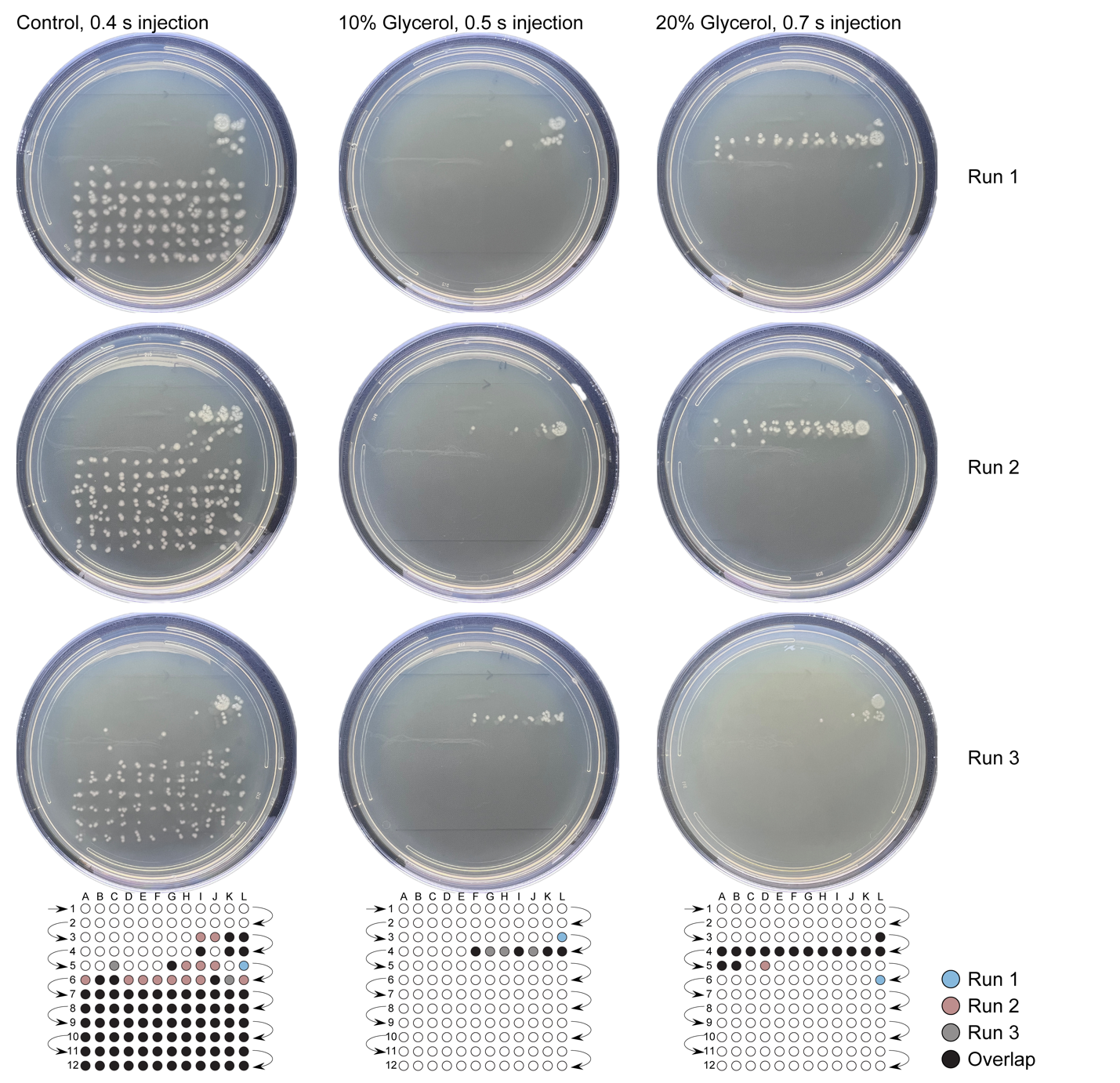
**

**Figure S3.** Images of *E. coli* B colonies after triplicate electrophoretic fractionation with injection time corrected for sample buffer viscosity. *E. coli* B were resuspended in sample buffer (control) and sample buffer supplemented with 10% or 20% glycerol. Injection times were varied based on viscosity of sample buffer to load approximately 900 cells per injection. Plates were incubated at 32 °C for 15 h and imaged. Reference grids are provided below each set of images to highlight the location of CFUs.

**
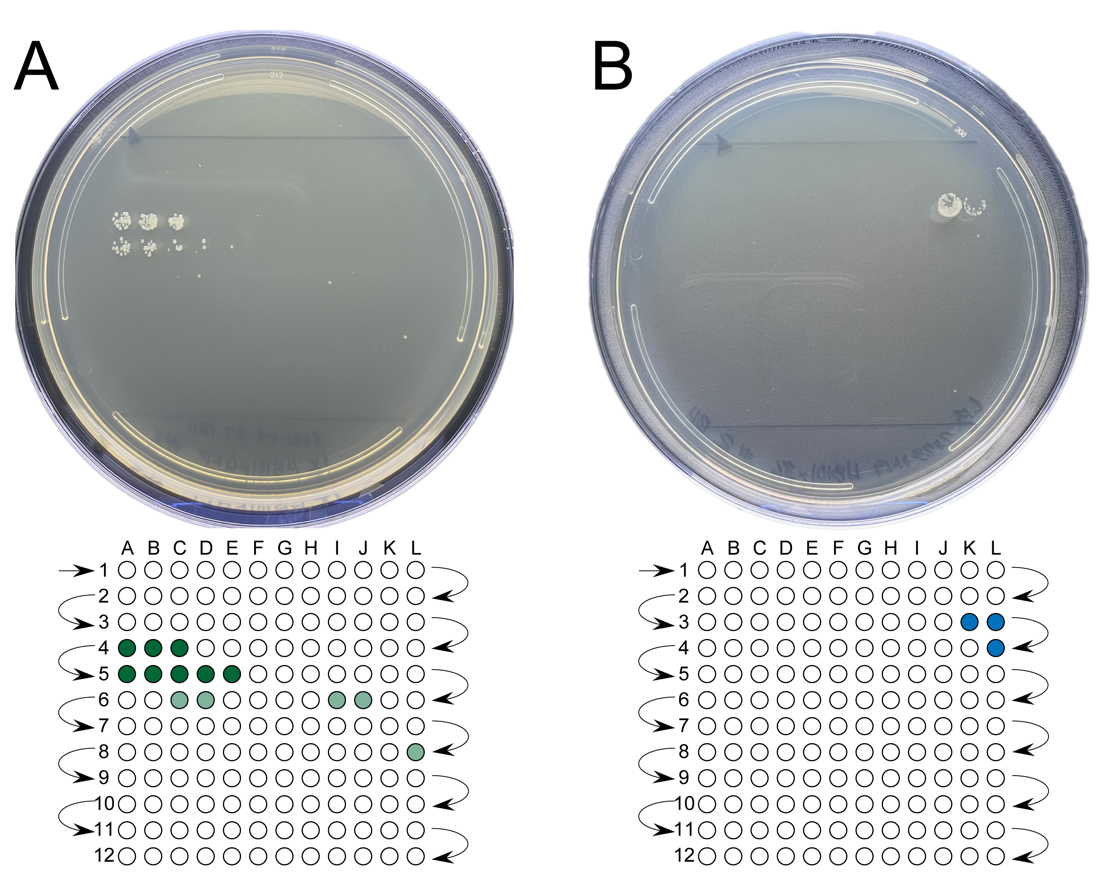
**

**Figure S4.** Comparison of *E. coli* HB101 colonies after electrophoretic fractionation using our previously reported protocol and our current protocol. A: 2,500 cells were loaded by hydrodynamic injection and collected onto LB agar (fraction width: 9.1 s). B: 50,000 cells were loaded by hydrodynamic injection and collected onto LB agar (fraction width: 4.1 s). Plates were incubated at 37 °C for 15 h and imaged. Reference grids are provided below each image to highlight the location of CFUs.
